# Epidemiological investigation of suspected autism in children and implications for healthcare system: a mainstream kindergarten-based population study in Longhua District, Shenzhen

**DOI:** 10.1186/s12887-015-0531-4

**Published:** 2015-12-15

**Authors:** Weikang Yang, Hui Xia, Guoming Wen, Li Liu, Xiaoyuan Fu, Junqiang Lu, Haitao Li

**Affiliations:** Center for Maternal and Child Healthcare of Longhua District, Shenzhen, China; Health Education Institute of Longhua District, Shenzhen, China; Health Inspection Institute of Longhua District, Shenzhen, China; School of Public Health, Sun Yat-Sen University, Guangzhou, China; School of Medicine, Shenzhen University, Nanhai Ave 3688, Shenzhen, Guangdong PR China

**Keywords:** Autism spectrum disorder, Developmental disorder, Prevalence, Risk factors, China

## Abstract

**Background:**

Individuals with autism put a heavy demand on medical services, and prevalence estimates are needed for the planning of such services. Screening for autism in children has important implications for individuals and policy makers. This study aimed to estimate prevalence of suspected autism in children in Longhua District, Shenzhen, and to investigate risk factors for autism.

**Methods:**

A cross-sectional study was conducted in Longhua District, Shenzhen in October 2014. A total of 141 kindergartens were approached and consented to participate in the current study. All children who met the inclusion criteria were screened for autism by using the Autism Behavior Checklist (ABC).

**Results:**

15,200 children in total completed the survey and were included in the final analysis. 2.6 % (95 % CI 2.3-2.9) respondents had a high probability of autism, while 4.0 % (95 % CI 3.7-4.3) respondents had questionable autism. Male children were more likely to develop autism when compared with their female counterparts (*P* < 0.001). Children of mothers with a lower education level and younger age tended to develop autism (*P* < 0.001).

**Conclusions:**

Our study shows a high prevalence rate of suspected autism in children which suggests an urgent need of early detection of autism with ABC across the Shenzhen city, or even around China. Further studies with diagnostic procedure are warranted. Maternal age and education level, and gender of children are possible factors related to autism.

## Background

Autism spectrum disorder (ASD) is a pervasive developmental disorder, which has three core symptoms including social barriers, language barriers and repetitive stereotyped behaviors and narrow interests [1]. According to previous versions of Diagnostic and Statistical Manual of Mental Disorders (DSM), ASD comprises of autistic disorder, Asperger syndrome and pervasive developmental disorders not otherwise specified [2]. In DSM-V, different types of ASD are combined in one broad category [3]. It was shown that the prevalence of autism among children aged 5 to 9 in the UK was 157 per 10000 in 2009 [4]. The prevalence of parent-reported diagnosis of ASD was 110 per 10000 among children aged between 3 to 17 years in the USA in 2007 [5]. In China, a national prevalence estimate of autism is lacking. A recent study by Sun et al. [6] reported that, in Beijing, the prevalence of ASD among children aged between 6 and 10 was about 1 % which is similar to that of developed counties. But, the reported prevalence of autism varied substantially across studies by gender, location of residence, date of publication, and source of sample.

Studies have well documented that affected individuals and their family members are substantially impacted by ASD both functionally and financially, for example a decrease in quality of life of family members due to stress caused by ASD, and an increase in parents’ working hours to meet financial needs of ASD [7]. Studies have revealed that impairments in socialization and communication skills continue as children with ASD enter adulthood [8]. Additionally, many children with ASD develop additional psychiatric symptoms and disorders in adolescence and adulthood [9]. Therefore, screening for autism in children has important implications for individuals and policy makers.

Shenzhen, situated in the Pearl River Delta region, is one of the most populous metropolitan areas in China. It has become one of the most important economic powerhouses in China, as well as the largest manufacturing base in the world, attracting millions of young migrant workers. According to the 2010 census, Shenzhen’s population is around 10.36 million with the average age about 30 years old [10]. Among the total, 9.8 % (about 1 million) are between the age of 0 ~ 14 years [10]. Individuals with autism put a heavy demand on medical services, and prevalence estimates are needed for the planning of such services. However, little research has been conducted to investigate the prevalence of autism among children in Shenzhen. This study aims to estimate the prevalence of suspected autism among children in Longhua District, Shenzhen, and to explore parental risk factors for autism.

## Methods

### Participants and procedures

A cross-sectional study was conducted in Longhua District, Shenzhen in October 2014. The target population consisted of, 1)all children born between, January 1, 2010 and December 31, 2010; 2)currently resided within the selected district, and 3)attended mainstream middle-class kindergartens in the selected district during the study period. Ethical approval for the study was granted by the Longhua District Center for Maternal and Child Healthcare Ethics Committee.

All kindergartens - a total of 141 - in the selected district were approached and consented to participate in the current study. We screened all children who met the inclusion criteria for autism. Data were collected by a parent-report questionnaire. School staff provided parents with an information sheet and consent form prior to screening evaluation. A total of 15,746 parents consented to participate, while 1510 rejected, with a response rate of 91.2 %.

### Measures

Presence or absence of suspected autism was the outcome variable of interest. The Autism Behavior Checklist (ABC) was used to identify potential autism cases. The adapted Chinese version of ABC had substantial validity and reliability for screening of autism [11]. ABC asked about characteristic autism behavior, which was applied to individuals aged between 18 months and 35 years. The Chinese version consisted of 57 items, including 11 items for relationships, 10 items for tapping sensory behaviors, 13 items for language, 12 items for the use of physical development, and 11 items for daily living skills. Each item rated between 1 and 4. The total score was generated by adding the scores for each item. A cut-off total score of >67 was used for indicating children with a high probability of autism, while a total score between 53 and 67 indicated questionable autism. Studies in China have shown good agreement between ABC (>67) and diagnostic criteria [12].

Some items of interest regarding parental socio-demographic characteristics were appended to the questionnaire. The information with respect to parental education level was collected. Education level referred to the highest academic qualification, and was collapsed into four categories including below middle school, high school and equivalent, 3-year college and 4-year university and above. The parental age at the time when the child was delivered was coded. Whether the child under screening had siblings was collected. Additionally, the children’s gender was documented.

### Statistical analysis

All data were input into Epidata database. Frequencies and percentages of children with suspected autism were presented. These figures were also stratified by children’s gender. All reported frequencies were un-weighted. Parental socio-demographic characteristics were described, and comparisons were made between the children with and without suspected autism by using Chi-square tests (or ANOVA where appropriate). Multiple logistic regression models were used to explore factors possibly associated with the development of autism. We constructed two models. The first one collapsed children into two categories: subjects with high probability of autism and the others, while the second one grouped individuals with high probability and questionable autism as one category. Confounders recruited in regression models included all variables collected in the current study. All analyses were performed using SPSS19.0, and a *P* value of less than 0.05 was adopted as the statistically significant level.

## Results

Missing data were serious (larger than 20 %) for 546 subjects (about 3.5 % of the total); these children were thus excluded from the final analysis. Missing values for the rest respondents on all variables were less than 10 %.

Of the 15,200 respondents, more were male (54.8 %). A total of 398 respondents reported ABC total scores higher than 67, whereas 611 respondents reported a score between 53 and 67. Two point six (95 % confidence internal [95 % CI] 2.3, 2.9) respondents had a high probability of autism, while 4.0 % (95 % CI 3.7, 4.3) respondents had questionable autism. Among male respondents, 3.2 % (95 % CI 2.8, 3.6) had a high probability of autism which was higher than that in female respondents (1.9 %, 95 % CI 1.6, 2.2). Similarly, for the respondents with questionable autism, the percentage was higher for male than for female individuals, 4.7 % (95 % CI 4.2, 5.2) and 3.2 % (95 % CI 2.8, 3.6) respectively (Table 1).

**Table 1:** Prevalence rate of suspected autism in children

Characteristics	Number (%) of respondents	Number (%) [95 % CI]	
		ABC total score: >67	ABC total score: 53 ~ 67
Gender			
Male	8319(54.8)	268(3.2)[2.8, 3.6]	390(4.7)[4.2, 5.2]
Female	6869(45.2)	130(1.9)[1.6, 2.2]	221(3.2)[2.8, 3.6]
Total	15188(100.0)	398(2.6)[2.3, 2.9]	611(4.0)[3.7, 4.3]

*ABC* autism behavior checklist

*CI* confidence interval

ABC total score >67 indicates a high probability of autism

ABC total score between 53 and 67 means questionable autism

Mothers of children with a high probability or questionable autism usually obtained a lower level of education when compared with mothers of children without suspected autism (*P* < 0.001). Similarly, fathers of children with a high probability or questionable autism tended to possess a lower education level (*P* < 0.001). Mothers of children with suspected autism (25.54 and 26.12 years for those with a high probability and questionable autism, respectively) were younger than those of children without suspected autism (27.17 years) (*P* < 0.001). Similar findings were observed for fathers. Children with suspected autism were more likely to have siblings than those without suspected autism (*P* < 0.001) (Table 2).

**Table 2:** Parental socio-demographic characteristics of children with and without suspected autism

Characteristics	With suspected autism		Without suspected autism	*P* value
	High probability	Questionable		
Maternal education				<0.001
Middle school and below	221(58.5)	286(49.3)	4046(29.5)	
High school and equivalent	100(26.5)	185(31.9)	4578(33.4)	
3-year college	40(10.6)	63(10.9)	2907(21.2)	
4-year university and above	17(4.5)	46(7.9)	2169(15.8)	
Paternal education				<0.001
Middle school and below	188(49.6)	216(37.4)	3149(23.2)	
High school and equivalent	104(27.4)	191(33.0)	3995(29.5)	
3-year college	48(12.7)	84(14.5)	3000(22.1)	
4-year university and above	39(10.3)	87(15.1)	3412(25.2)	
Maternal age^a^(mean, SD)	366(25.54)	26.21(4.412)	27.17(4.097)	<0.001
Paternal age^a^(mean, SD)	28.22(5.235)	28.68(4.492)	29.58(4.568)	<0.001
Whether the only child				<0.001
Yes	140(41.7)	222(43.1)	6292(50.2)	
No	196(58.3)	293(56.9)	62332(49.8)	

*SD* standard error

^a^Parental age refers to the time when the child was delivered

By using multiple logistic regression models, we explored factors possibly related to the development of autism. Table 3 shows that male children were more likely to develop autism when compared with their female counterparts (*P* < 0.001). Children of mothers with a lower education level tended to develop autism than those of mothers with a higher education level (*P* < 0.001). Maternal age was found negatively associated with the development of autism (*P* < 0.001). However, paternal education level, paternal age and whether the child has siblings were not found statistically associated with the development of autism (Table 3).

**Table 3:** Factors associated with autism in children

Factors	Model 1		Model 2	
	OR (95 % CI)	*P* value	OR (95 % CI)	*P* value
Gender				
Female	ref		ref	
Male	1.596(1.244,2.021)	<0.001	1.491(1.280,1.738)	<0.001
Maternal education				
4-year university and above	ref		ref	
3-year college	1.674(0.795,3.524)	0.175	1.235(0.839,1.819)	0.284
High school and equivalent	3.051(1.492,6.240)	0.002	2.090(1.437,3.038)	<0.001
Middle school and below	5.511(2.612,11.630)	<0.001	3.554(2.381,5.303)	<0.001
Paternal education				
4-year university and above	ref		ref	
3-year college	0.960(0.576,1.599)	0.874	0.929(0.689,1.253)	0.630
High school and equivalent	1.064(0.652,1.737)	0.803	1.081(0.808,1.445)	0.600
Middle school and below	1.672(0.999,2.797)	0.050	1.336(0.974,1.832)	0.072
Maternal age^a^(mean, SD)	0.921(0.884,0.961)	<0.001	0.948(0.923,0.974)	<0.001
Paternal age^a^(mean, SD)	1.022(0.986,1.059)	0.232	1.003(0.980,1.027)	0.784
Whether the only child				
Yes	ref		ref	
No	1.082(0.842,1.390)	0.539	1.014(0.863,1.192)	0.863

*SD* standard error

*CI* confidence interval

*OR* odds ratio

*Ref* reference group

^a^Parental age refers to the time when the child under screening was delivered

Model 1: collapsed individuals into two categories: subjects with high probability and the others

Model 2: grouped individuals with high probability and questionable autism as one category

## Discussion

Our study estimated the prevalence of suspected autism in children attending mainstream middle-class kindergartens by using a screening procedure, which suggests a high prevalence rate of suspected autism in Longhua District, Shenzhen. Our study identified three factors possibly associated with the development of autism, including maternal education level, gender of child, as well as maternal age.

Results showed that the prevalence of suspected autism was 6.6 % which is much higher than that reported by previous studies in China. A systematic review conducted by Sun et al. [13] showed that the prevalence of childhood autism in China ranged between 0.32 per 10,000 and 29.5 per 10,000. Possible explanations of this observation include varying study locations, population of study as well as adoption of different screening instruments. China is vast in area and varied in culture. The recognition of autism features in one culture may be different from another, since each culture has a set of specific behavioral norms and expectations, which are usually different from the others [14]. Most of previous studies estimated prevalence rate based on samples from the stratified general population, while the present study screened all children attending mainstream middle-class kindergartens. Additionally, most of previous studies included children aged between 2 and 3 years. Since the average age of diagnosis of autism is suggested to be 41 to 60 months [15], the higher prevalence rate of the current study than that of previous ones is expected. It has been well documented that the prevalence of autism was strongly associated with the choice of screening instrument [16]. The utilization of different screening instrument might thus be another possible factor contributing to gap in the estimation of prevalence rate of autism between our study and previous ones.

Our prevalence estimate is also higher than that reported by Western studies, although studies have shown that the prevalence of autism has increased during the past several decades in Western countries. The Centers for Disease Control’s (CDC) Autism and Developmental Disabilities Monitoring (ADDM) Network showed a 78 % increase in ASD prevalence in the United States between 2002 and 2008 [17]. A recent estimate by the CDC revealed that the prevalence of parent-reported ASD among children aged 6-17 may be as high as 2 % in 2011-2012 [18]. A study by Atladottir et al. [19] compared the time trend of reported diagnosis of ASD in Denmark, Finland, Sweden and Western Australia, which showed an increase in age-specific prevalence of reported diagnosis of ASD across the four countries. Un-implementation of diagnosis procedure of the current study might be one possible explanation of this observation. Our study did not use any diagnostic instrument for confirmation of autism. Although the ABC questionnaire was found to be valid for screening of autism, the absence of diagnosis procedure may lead to an overestimate of the current study. It should be cautious when comparison was made between the current study and Western studies.

Results showed that male children with autism outnumbered females in Shenzhen with a male: female ratio of 1.7:1, which is consistent with previous epidemiological studies. Fombonne [20] performed a literature review and found that male: female ratios ranged between 1.33:1 and 16.0:1. Up to now, a definitive explanation has not been reached with respect to sex-specific factors that may increase male children’s risks for, or protect female children from autism. The finding, that the rate of suspected autism was much higher for children of less educated mothers, is similar to some previous studies. A study conducted in Sweden by Rai et al. [21] showed that mothers with lower level of education tended to have a higher risk of having children diagnosed with ASD. Fujiwara [22] also indicated that toddlers of mothers with lower levels of education had greater rates of suspected ASD. This may be because mothers with lower levels of education are more likely to be exposed to risk factors for ASD during pregnancy or infancy such as smoking [23] and insufficient micronutrients [24].

It was shown that maternal age at delivery was negatively associated with risk of autism. The current literature showed mixed views regarding the relationship between maternal age and autism. The study by Croen et al. [25] showed that risk for autism increased as maternal age increased. A cohort study by Golding et al. [26] also showed that older motherhood was associated with ASD diagnosis. However, a previous study using a cohort of Chinese children showed that maternal age was not statistically significantly associated with the development of autism [27]. The conflict findings across different studies warrant further investigations.

Limitations of the study should be addressed. Firstly, the generalizability of study findings is limited. For one thing, the study participants were selected from just one district of Shenzhen city. For another, we only screened children attending mainstream middle-class kindergartens, while many diagnosed cases may be attending special schools. Therefore, findings of the current study cannot be generalized not only to children across the entire city, but also to those in general. Secondly, case identification procedures may bias our prevalence estimates. We did not perform case diagnostic procedure but only used ABC which is a screening tool to detect cases. Therefore, the 6.6 % of children with suspected autism (i.e., reported ABC total score higher than 53) suggesting a large proportion of false positive, even among those with high probability of autism (i.e., reported ABC total score higher than 67). Future studies with accepted gold standard diagnostic assessment are needed for better estimate of prevalence of autism. Thirdly, other factors not included in the current study may bias our findings. Socioeconomic factors of parents such as occupation and household income were not collected in the current study. Children characteristics including genetics and intellectual disability et al were also not collected. These confounders were thus not included in the multiple regression analysis. Fourthly, the cross-sectional nature of our study prevents us from establishing causal relationships between maternal education and age and risk of autism.

## Conclusions

We showed a high prevalence of suspected autism in Longhua District, Shenzhen which implies a big challenge faced by affected families and healthcare system. Our findings suggest an urgent need of early detection of autism with ABC across the Shenzhen city, or even around China since screening is important in early identification which facilitates subsequent diagnostic assessment, access to intervention and services and, ultimately, long-term improvements of children with autism. To improve awareness of autism among Chinese parents, and effectiveness of intensive early interventions in long-term improvements is one possible strategy for early detection and diagnosis of autism. Formulation and dissemination of autism diagnosis and management guidelines – like hypertension management guidelines – are essential for autism diagnosis and access to interventions, at last, for improved prognosis of autism of children at risk. The National Autism Plan for Children of UK may shed light on autism management in Shenzhen and China.
